# *Lactobacillus plantarum*-derived indole-3-lactic acid inhibits prostate cancer progression through ASF1B/ENO1 axis and remodels the tumor microenvironment to enhance anti-PD-1 therapy

**DOI:** 10.1186/s43556-026-00540-2

**Published:** 2026-08-06

**Authors:** Zhengshi Wang, Chengyou Jia, Yongqiang Liu, Youlutuziayi Rixiati, Wentao Zhang, Shiyu Mao, Haotian Chen, Libin Zou, Chen Ye, Bing Shen, Xudong Yao

**Affiliations:** 1 Department of Urology, Shanghai Tenth People’s Hospital, School of Medicine, Tongji University, Shanghai, China; 2 School of Medicine, Urologic Cancer Institute, Tongji University, Shanghai, China; 3 Department of Breast and Thyroid Surgery, Shanghai Tenth People’s Hospital, School of Medicine, Tongji University, Shanghai, China; 4 Shanghai Center of Thyroid Diseases, Shanghai Tenth People’s Hospital, School of Medicine, Tongji University, Shanghai, China; 5 Department of Nuclear Medicine, Shanghai Tenth People’s Hospital, School of Medicine, Tongji University, Shanghai, China; 6 Department of Pathology, Xinhua Hospital Affiliated to Shanghai Jiaotong University School of Medicine, Shanghai, China; 7 Department of Urology, Changhai Hospital, Naval Medical University, Shanghai, China; 8 Tongji University Cancer Center, Shanghai Tenth People’s Hospital, School of Medicine, Tongji University, Shanghai, China

**Keywords:** Prostate cancer, Indole-3-lactic acid, *Lactobacillus plantarum*, Tumor microenvironment

## Abstract

Prostate cancer (PCa) is one of the most common cancers in males, and its treatment remains challenging due to the tumor microenvironment (TME) with immunosuppressive properties and limited response to anti-PD-1 therapy. Gut microbiota-derived metabolites have recently emerged as modulators of cancer immunometabolism, however, their role in PCa progression and immunotherapy is poorly understood. Here we found that indole-3-lactic acid (ILA), a metabolite produced by *Lactobacillus plantarum*, exerted dual anti-tumor effects on PCa cells and the TME. Mechanistically, ILA activates the aryl hydrocarbon receptor (AHR), and the resulting AHR/ARNT heterodimer translocates into the nucleus and binds to the promoter of ASF1B. This heterodimer then recruits the HDAC1/2-NuRD complex to reduce H3K27ac levels and suppress ASF1B expression. ASF1B binds to specific residues of ENO1 via its N-terminal core domain and enhances ENO1 enzymatic activity. ILA-induced downregulation of ASF1B impairs this interaction, reduces ENO1 activity, and suppresses the PI3K/Akt pathway, thereby inhibiting the malignant phenotypes of PCa cells. Concurrently, ILA decreased CXCL8 secretion by inhibiting the PI3K/Akt/NF-κB pathway, enhancing CD8^+^ T cell infiltration and M1 macrophage polarization, thereby remodeling the TME. Additionally, ILA synergized with anti-PD-1 therapy to more effectively suppress tumor growth. These findings reveal a novel mechanism by which gut microbiota-derived metabolites regulate PCa progression and immunometabolism, positioning ILA as a potential therapeutic agent to improve PCa treatment.

**Supplementary Information:**

The online version contains supplementary material available at 10.1186/s43556-026-00540-2.

## Introduction

Prostate cancer (PCa) is one of the most common cancers in males, with both incidence and mortality continuing to rise worldwide. According to GLOBOCAN 2022, 1.47 million new cases of PCa are diagnosed annually, resulting in approximately 397,000 deaths globally [1]. The five-year survival rate for patients with early-stage PCa is favorable; however, outcomes remain poor for those with advanced disease, despite multiple therapeutic strategies, including surgery and androgen deprivation therapy (ADT) [2–5]. Therefore, the development of novel therapeutic strategies is urgently needed to improve clinical outcomes in patients with PCa.

Studies have shown that the pronounced immunosuppressive features of PCa arise from multiple mechanisms. First, reduced infiltration of cytotoxic CD8^+^ T cells is a key determinant and is strongly associated with poor prognosis in patients with PCa [6]. Second, regulatory T cells (Tregs) together with myeloid-derived suppressor cells (MDSCs) inhibit the activation and proliferation of CD8^+^ T cells through the secretion of immunosuppressive cytokines such as TGF-β and IL-10, thereby attenuating antitumor immune responses [7, 8]. In PTEN-deficient PCa, upregulation of IDO1 is associated with increased infiltration of FoxP3^+^ Tregs, further promoting the establishment of an immunosuppressive microenvironment [9]. Third, tumor-associated macrophages (TAMs), another major component of the tumor microenvironment (TME), display distinct phenotypes; among them, M2-like macrophages promote immune suppression by enhancing the production of anti-inflammatory factors [10]. In addition, overexpression of immune checkpoint molecules, such as PD-1 and PD-L1, is frequently observed in PCa and contributes to tumor immune evasion by inhibiting T cell activation and proliferation [11, 12]. Together, these factors collectively shape the immunosuppressive TME of PCa, which not only promotes tumor progression but also presents a major challenge for current therapeutic strategies.

Accumulating evidence shows that gut microbiota (GM) and their metabolites modulate tumor development and anti-tumor immunity [13–17]. The possibility of intervening in tumor-associated processes by manipulating GM and their metabolites has thus recently emerged as a viable therapeutic approach for numerous malignancies. Nevertheless, their exact functions in PCa have not yet been clarified.

In the present study, using 16S rRNA sequencing, in vitro functional experiments and in vivo tumor models, we found that indole-3-lactic acid (ILA), a metabolite derived from *Lactobacillus plantarum* (*L. plantarum*), not only directly inhibited the malignant biological behaviors of PCa cells but also suppressed PCa progression by remodeling the TME.

## Results

### Relative abundance of the Lactobacillaceae family was higher in early-stage PCa

**Fig. 1:**
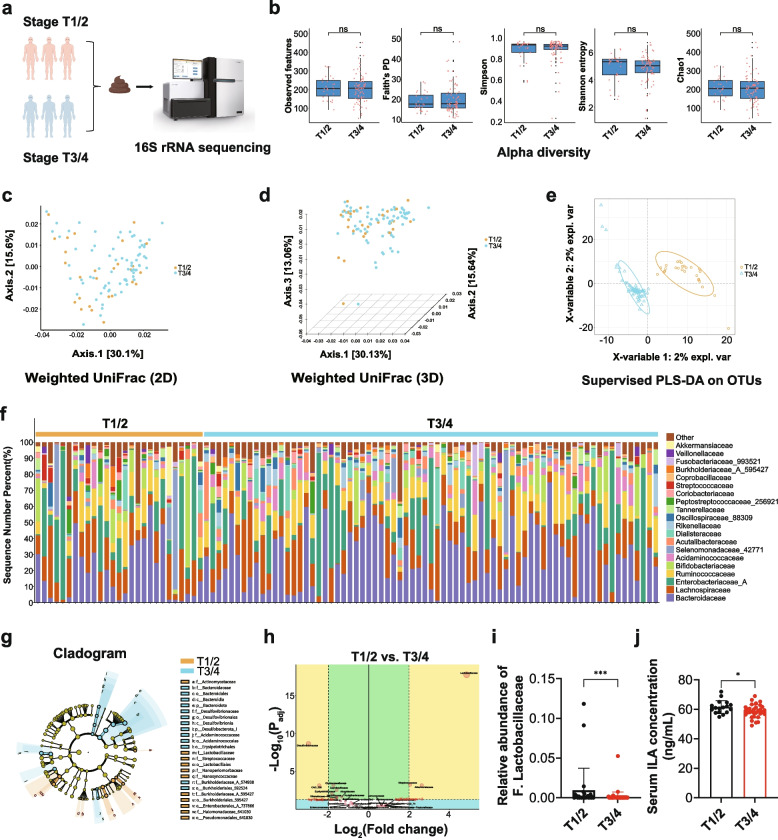
Fecal 16S rRNA sequencing analysis. **a** Flow chart of 16S rRNA sequencing (T1/2: *n* = 27; T3/4: *n* = 73). **b** Alpha-diversity index analysis. **c** 2D principal coordinate analysis scatter plot. **d** 3D principal coordinate analysis scatter plot. **e** Supervised PLS-DA score plot. **f** Relative abundance of bacteria at the family level. **g** Cladogram showing the phylogenetic distribution of microbial biomarkers between stage T1/2 and T3/4 identified by LEfSe analysis. **h** Volcano plot of differentially abundant microbial taxa between stage T1/2 and T3/4. **i** Comparison of the relative abundance of the Lactobacillaceae family between stage T1/2 and T3/4. **j** Comparison of serum ILA concentrations between stage T1/2 and T3/4 (*n* = 49). Statistical comparisons were performed using Student’s t-test for two groups. ILA, indole-3-lactic acid; LEfSe, linear discriminant analysis effect size; PLS-DA, partial least squares–based discriminant analysis; ns, no statistical difference

A total of 100 patients with PCa who underwent surgery were included in this study (Table S1). The cohort was divided into two groups according to T stage (T1/2, early stage, *n* = 27; T3/4, advanced stage, *n* = 73), and fecal 16S rRNA sequencing was performed for each group (Fig. 1a). Multiple α-diversity indices were used to comprehensively evaluate microbial diversity, and no significant differences were observed between the T1/2 and T3/4 groups (Fig. 1b, *p* > 0.05). Although unsupervised principal coordinate analysis (PCoA) revealed substantial overlap in the overall microbial community structure between the two groups, supervised partial least squares-based discriminant analysis (PLS-DA) achieved clear separation, indicating the presence of group-specific microbial abundance patterns (Fig. 1c-e).

Stacked bar plots of per-sample relative abundance showed that, despite broadly similar community structures between the T1/2 and T3/4 groups, differences in the relative abundance of several bacterial families were evident (Fig. 1f). Cladogram analysis further indicated that the Actinomycetaceae and Lactobacillaceae families were significantly enriched in fecal samples from patients with stage T1/2 disease (Fig. 1g). Consistently, volcano plot analysis and intergroup differential testing demonstrated that the Lactobacillaceae family represented the most significantly enriched and differentially abundant bacterial taxon in the T1/2 group (Fig. 1h and i, *p* < 0.001).

Based on the 16S rRNA sequencing results, we found that patients with early-stage PCa (T1/2) had a considerably greater relative abundance of the Lactobacillaceae family. *L. plantarum*, a common member of the Lactobacillaceae family, has been implicated in the progression of colorectal cancer [18–20]. We further quantified the relative abundance of *L. plantarum* in fecal samples from PCa patients and verified that its level was significantly higher in the T1/2 group than in the T3/4 group (Fig. S1a). Additionally, subgroup analysis stratified by Gleason score revealed that patients with low Gleason scores (3 + 3, 3 + 4) exhibited markedly higher *L. plantarum* abundance compared with those with high Gleason scores (≥ 4 + 3) (Fig. S1b). Because ILA, the major metabolite of *L. plantarum*, can be absorbed into the bloodstream [21], we hypothesized that it might influence tumors outside the gastrointestinal tract through systemic circulation. To preliminarily explore this possibility, we measured serum ILA concentrations in PCa patients and found significantly higher levels in the T1/2 group than in the T3/4 group (Fig. 1j). Consistently, serum ILA levels were also significantly higher in patients with low Gleason scores than in those with high Gleason scores (Fig. S1c). Therefore, we focused on ILA for subsequent analyses.

### ILA inhibited the malignant biological behaviors of PCa cells

**Fig. 2:**
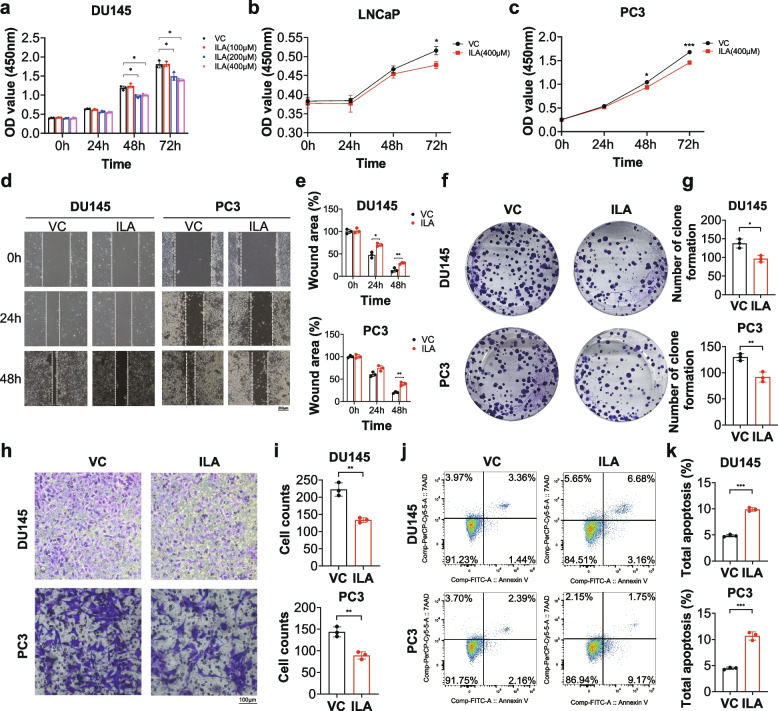
ILA inhibited the malignant biological behaviors of PCa cells. **a** Effect of ILA at various concentrations on DU145 cell proliferation assessed by CCK8 assay. **b** LNCaP cell proliferation measured by CCK8 assay. **c** PC3 cell proliferation measured by CCK8 assay. **d** Wound-healing assay of DU145 and PC3 cells. White dashed lines indicate scratch edges. **e** Quantification of Fig. 2d. **f** Plate clone formation assay of DU145 and PC3 cells. **g** Quantification of Fig. 2f. **h** Transwell invasion assay of DU145 and PC3 cells. **i** Quantification of Fig. 2h. **j** Apoptosis assay of DU145 and PC3 cells. **k** Quantification of Fig. 2j. Each experiment was repeated three times. Data are expressed as mean ± SD (*n* = 3). Statistical comparisons were performed using Student’s t-test for two groups and one-way ANOVA followed by Tukey’s post hoc test for multiple groups. OD, optical density

Given that the highest concentration of ILA in dimethyl sulfoxide (DMSO) (stock solution) was 487.31 mM, the maximum working concentration was set at 400 μM. ILA was therefore applied at doses of 0, 100 μM, 200 μM, and 400 μM. As shown in Fig. 2a, ILA showed the strongest inhibitory impact on the growth of the DU145 PCa cell line at 400 μM, which was selected as the optimal concentration for subsequent experiments. Cell counting kit-8 (CCK8) assays performed in two additional commonly used PCa cell lines, PC3 and LNCaP, further demonstrated that ILA significantly suppressed cell growth (Fig. 2b and c). Given the relatively weaker inhibitory effect observed in LNCaP cells, DU145 and PC3 cell lines were selected for subsequent in vitro experiments.

Wound-healing assays, plate clone formation assays, and Transwell invasion assays collectively showed that ILA markedly inhibited the migratory capacity, clonogenic potential, and invasive ability of PCa cells (Fig. 2d-i, *p* < 0.05). Apoptosis assays further revealed that ILA significantly promoted apoptosis in PCa cells (Fig. 2j and k, *p* < 0.01). Together, these findings indicate that ILA effectively suppresses the malignant biological behaviors of PCa cells.

### ILA suppressed ASF1B expression through H3K27ac deacetylation

**Fig. 3:**
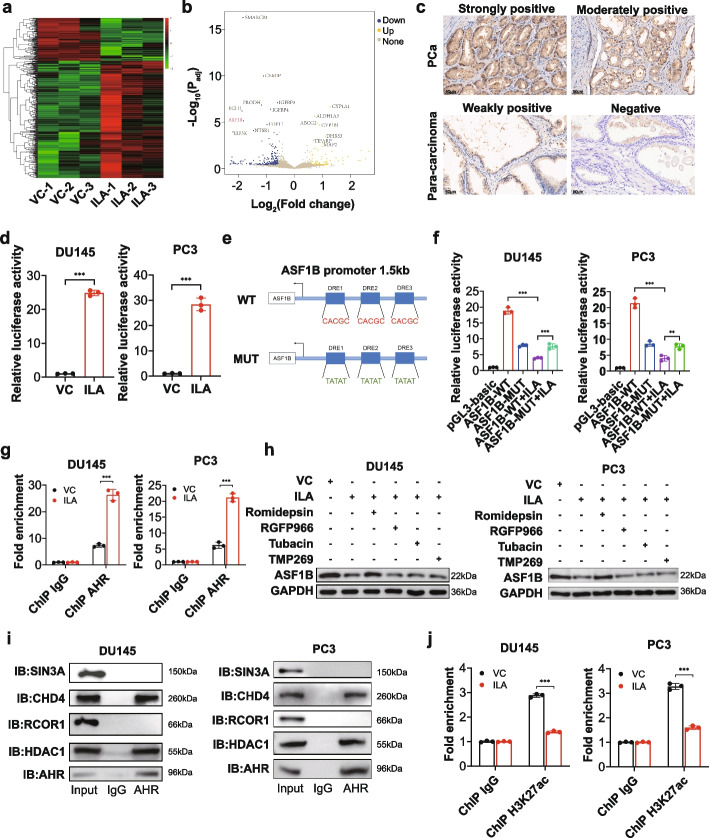
ILA suppressed ASF1B expression through H3K27ac deacetylation. **a** Heat map showing differentially expressed genes between the ILA-treatment and VC groups. **b** Volcano plot showing up- and down-regulated genes. **c** Representative IHC images of ASF1B expression in PCa and para-carcinoma tissues. **d** Luciferase activity in PCa cells after ILA treatment. **e** Schematic diagram of WT and MUT DRE sequences in the ASF1B promoter. **f** Luciferase assay analyzing point mutations in the DRE. **g** ChIP-qPCR results showing enrichment of AHR at DRE sites. **h** Effect of ILA on ASF1B protein expression with different HDAC inhibitors: Romidepsin (5 nM), RGFP966 (5 μM), Tubacin (5 μM), TMP269 (10 μM). **i** Co-IP assays identifying potential corepressor complexes. **j** ChIP-qPCR results showing enrichment of H3K27ac at DRE sites. Each experiment was repeated three times. Data are expressed as mean ± SD (*n* = 3). Statistical comparisons were performed using Student’s t-test for two groups and one-way ANOVA followed by Tukey’s post hoc test for multiple groups. DRE, dioxin response element; AHR, aryl hydrocarbon receptor; HDAC, histone deacetylase; WT, wild type; MUT, mutant

To identify downstream targets of ILA, PC3 cells were subjected to RNA sequencing (RNA-seq) analysis after ILA administration. In comparison to the vehicle control (VC) group, 105 genes were upregulated and 287 genes were downregulated (Fig. 3a and b, *p* < 0.05).

The online Gene Expression Profiling Interactive Analysis (GEPIA) database was used to evaluate the differential expression and prognostic significance of the top 10 candidate genes. Among these, ASF1B was the only gene showing significantly elevated expression in PCa (Fig. S1d, *p* < 0.05) and was significantly correlated with both overall survival (OS) and disease-free survival (DFS) (Fig. S1e and 1f, *p* < 0.05). The reduction in ASF1B expression following ILA treatment was further confirmed by quantitative reverse transcription-polymerase chain reaction (qRT-PCR) (Fig. S2a, *p* < 0.001). Immunohistochemistry (IHC) staining revealed that ASF1B expression was predominantly moderate to strong in PCa tissues, whereas it was mostly weak or negative in para-carcinoma tissues (Fig. 3c). Western blot (WB) analysis further confirmed elevated ASF1B protein levels in PCa tissues (Fig. S2b). Collectively, these data indicate that ASF1B serves as a critical downstream target of ILA.

ILA is a well-established ligand of the aryl hydrocarbon receptor (AHR) [22]. To determine whether ILA activates AHR signaling in PCa cells, PC3-Lucia AHR and DU145-Lucia AHR reporter cell lines were generated. Luciferase reporter assays showed that ILA treatment markedly increased luciferase activity (Fig. 3d, *p* < 0.001). Antagonizing AHR via CH223191, a selective and specific AHR inhibitor, substantially attenuated the ILA-induced inhibition on the malignant behaviors of PCa cells, indicating that the tumor-suppressive function of ILA is dependent on AHR (Fig. S2c-2e). Previous studies have shown that upon activation, AHR underwent a conformational change that exposed its N-terminal nuclear localization signal. After translocation into the nucleus, AHR bound to the aryl hydrocarbon receptor nuclear translocator (ARNT) to form a heterodimer (AHR/ARNT), which recognized and bound to dioxin response elements (DREs, 5'-CACGC-3' or 5'-GCGTG-3') [23] (Fig. S3a). Analysis of National Center for Biotechnology Information (NCBI) database revealed three putative DRE sites within the promoter region of ASF1B (Fig. S3b). Consistently, Chromatin Immunoprecipitation Sequencing (ChIP-seq) data from the Cistrome DB showed a clear enrichment peak within the ASF1B promoter region (Fig. S3c). Interestingly, an H3K27ac enrichment peak was also detected at the same site (Fig. S3c). Because H3K27ac is typically associated with increased chromatin accessibility and transcriptional activation [24], and histone deacetylases (HDACs) can be recruited to DREs [25], we hypothesized that the AHR/ARNT heterodimer suppresses ASF1B transcription by recruiting HDACs to reduce H3K27ac levels at its promoter. To test this hypothesis, the DRE sequence within the ASF1B promoter was mutated and dual-luciferase reporter assays were performed (Fig. 3e). ILA treatment significantly suppressed ASF1B promoter activity in PCa cells (Fig. 3f). However, mutation of the DRE sequence abolished this inhibitory effect, as promoter activity remained comparable regardless of ILA treatment (Fig. 3f). ChIP-qPCR analysis further demonstrated that AHR enrichment at the DRE site increased significantly following ILA stimulation (Fig. 3g), indicating that ILA could reduce ASF1B transcription by activating AHR and promoting its binding to the DRE sequence.

We next investigated which HDAC family members were involved. Using whole-cell lysates, inhibitors targeting common HDACs (HDAC1–7) were tested. Treatment with Romidepsin, an HDAC1/2 inhibitor, significantly restored ASF1B expression. In contrast, inhibition of HDAC3 (RGFP966), HDAC6 (Tubacin), or HDAC4/5/7 (TMP269) did not markedly affect ASF1B levels (Fig. 3h). Because HDACs typically function within multiprotein corepressor complexes, we further performed co-immunoprecipitation (Co-IP) assays to examine three major HDAC-containing complexes (NuRD, SIN3A, and CoREST) [26]. The results showed that AHR interacted strongly with HDAC1 and CHD4 (a core subunit of the NuRD complex), whereas no interaction was detected with RCOR1 (a core subunit of the CoREST complex) or SIN3A (a core subunit of the SIN3A complex) (Fig. 3i). ChIP-qPCR analysis further demonstrated that ILA treatment reduced H3K27ac levels at the ASF1B promoter region (Fig. 3j). Together, these findings demonstrated that ILA activated AHR, promoting the formation of the AHR/ARNT heterodimer that bound to DRE sites within the ASF1B promoter. This interaction recruited the HDAC1/2-NuRD corepressor complex, leading to H3K27ac deacetylation and transcriptional repression of ASF1B.

### ILA and ASF1B knockdown exerted synergistic suppression effects

**Fig. 4:**
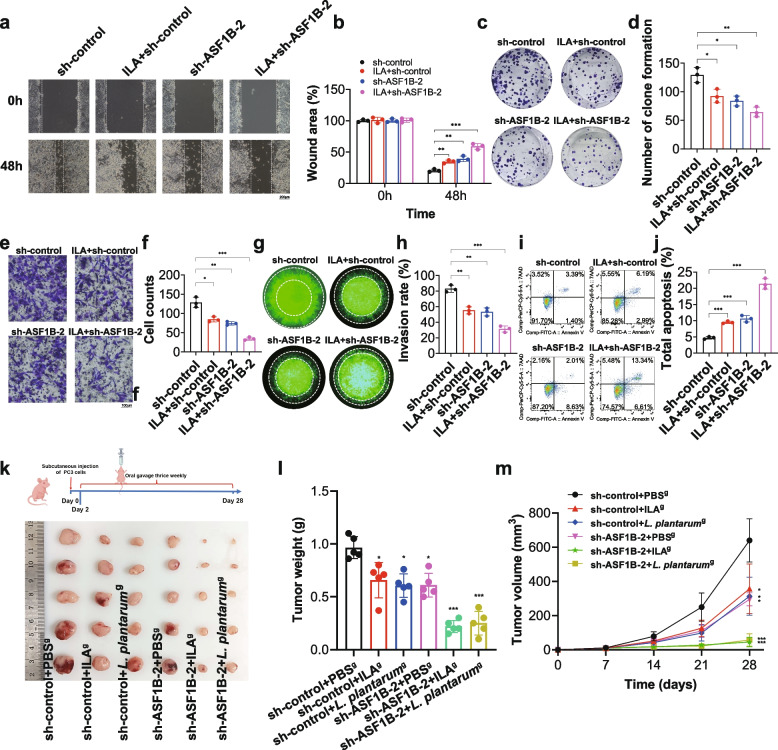
ILA and ASF1B knockdown exerted synergistic suppression effects. **a** Wound-healing assay. **b** Quantification of Fig. 4a. **c** Plate clone formation assay. **d** Quantification of Fig. 4c. **e** Transwell invasion assay. **f** Quantification of Fig. 4e. **g** 3D spheroid invasion assay. Inner dashed white circle indicates initial spheroid outline, and outer dashed circle indicates maximal invasion area after 120 h. **h** Quantification of Fig. 4g. **i** Apoptosis assay. **j** Quantification of Fig. 4i.** k** Nude mouse subcutaneous xenograft tumor models. ^g^gavage. **l** Tumor weight of Fig. 4k at the endpoint. ^g^gavage. **m** Changes in tumor volume (mm^3^) in each group. ^g^gavage. Each experiment was repeated three times. Data are expressed as mean ± SD (*n* = 3–5). Statistical comparisons were performed using Student’s t-test for two groups and one-way ANOVA followed by Tukey’s post hoc test for multiple groups. ILA, indole-3-lactic acid; ASF1B, anti-silencing function 1B; 3D, three-dimensional

Given the high expression of ASF1B in PCa cells, stable ASF1B-knockdown PC3 cell lines were generated using a short hairpin RNA targeting ASF1B (sh-ASF1B-2) (Fig. S4a). Wound-healing assays, plate clone formation assays, Transwell invasion assays, and three-dimensional (3D) spheroid invasion assays demonstrated that both ILA treatment and ASF1B knockdown markedly suppressed the migratory capacity, clonogenic potential, and invasive ability of PCa cells (Fig. 4a-h). Notably, the combination of ILA treatment and ASF1B knockdown produced a stronger inhibitory effect, indicating synergistic suppression (Fig. 4a-h). Consistently, both ILA and sh-ASF1B significantly increased apoptosis in PCa cells, and the combination further enhanced this effect (Fig. 4i and j, *p* < 0.01). To exclude potential off-target effects, additional functional assays were performed in DU145 cells using an independent shRNA (sh-ASF1B-1). The results confirmed that both ILA treatment and ASF1B knockdown significantly inhibited the malignant phenotype of PCa cells (Fig. S4b-4d). Furthermore, overexpression of ASF1B effectively reversed the antitumor effects induced by ILA.

Subcutaneous xenograft models in nude mice further demonstrated that *L. plantarum*, ILA, and sh-ASF1B each significantly inhibited tumor growth in vivo (Fig. 4k-m, Fig. S5a and 5b). Combined treatment with ILA or *L. plantarum* together with sh-ASF1B resulted in even greater tumor suppression. Collectively, these findings indicate that ILA and ASF1B knockdown synergistically inhibit PCa progression.

### ILA-induced downregulation of ASF1B inhibited PI3K/Akt signaling via ENO1

**Fig. 5:**
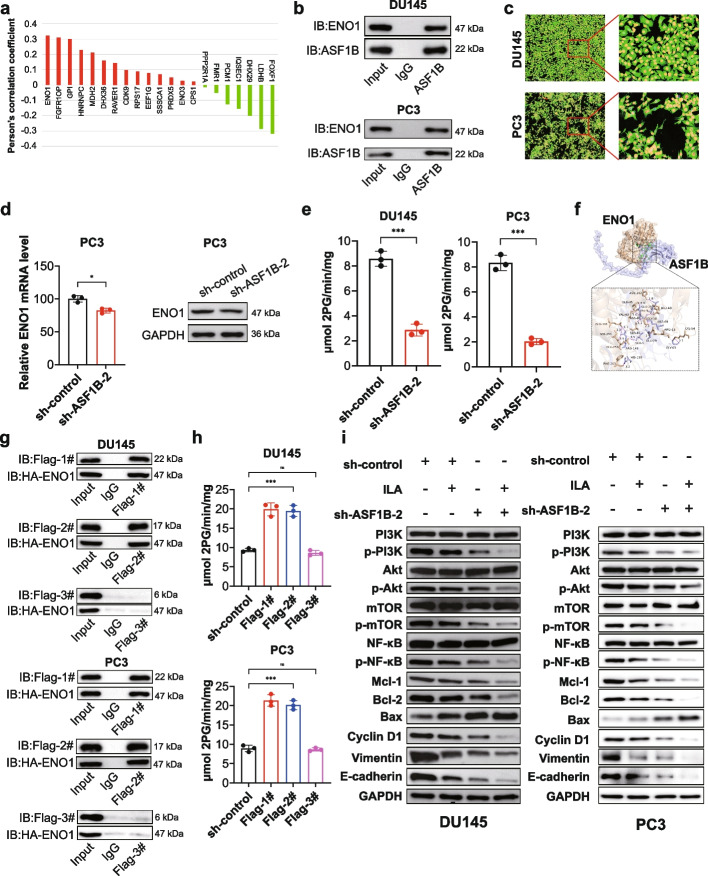
ILA-induced downregulation of ASF1B inhibited PI3K/Akt signaling via ENO1. **a** Overlap between ASF1B-interacting proteins (LC–MS) and ASF1B-co-expressed genes. **b** Co-IP assays confirming interaction between ASF1B and ENO1. **c** Immunofluorescence colocalization of ASF1B and ENO1. Red: ASF1B; green: ENO1; blue: DAPI. **d** Transcriptional and protein changes of ENO1 following ASF1B knockdown. **e** Changes in ENO1 enzymatic activity after ASF1B knockdown. **f** 3D molecular docking between ASF1B and ENO1. **g** Identification of ASF1B domains responsible for ENO1 interaction: Flag-1#, full-length; Flag-2#, N-terminal fragment (1–156 aa); Flag-3#, C-terminal fragment (156–202 aa). **h** Quantification of Fig. 5g. **i** Impact of ILA/ASF1B on PI3K/Akt pathway and key regulators. Each experiment was repeated three times. Data are expressed as mean ± SD (*n* = 3). Statistical comparisons were performed using Student’s t-test for two groups and one-way ANOVA followed by Tukey’s post hoc test for multiple groups. LC–MS, liquid chromatography–mass spectrometry; Co-IP, co-immunoprecipitation

To identify direct downstream targets of ASF1B, we analyzed liquid chromatography-mass spectrometry (LC–MS) data reported by Liu et al. [27] (Table S2). Combined with correlation analysis (Table S3), ASF1B and ENO1 showed the strongest correlation and a predicted direct interaction (Fig. 5a). Co-IP assays confirmed a direct interaction between ASF1B and ENO1 (Fig. 5b), and immunofluorescence analysis further demonstrated that ASF1B and ENO1 predominantly colocalize in the nucleus (Fig. 5c, Fig. S5c). We next investigated the impact of ASF1B knockdown on ENO1 expression and found a modest decrease in ENO1 expression at both the transcriptional and translational levels (Fig. 5d). Analysis of GEPIA dataset indicated that ENO1 mRNA expression did not differ significantly between PCa and normal tissues (Fig. S5d) and was not associated with patient prognosis (Fig. S5e). These findings suggest that ASF1B may not exert its primary effects through regulation of ENO1 expression levels. Because ENO1 is a member of the enolase enzyme family [28], we next assessed whether ASF1B influences ENO1 enzymatic activity. Knockdown of ASF1B markedly reduced ENO1 enzymatic activity (Fig. 5e, *p* < 0.001). Functional assays further showed that pharmacological inhibition of ENO1 activity using AP-III-a4 significantly suppressed PCa cell proliferation and invasion while promoting apoptosis (Fig. S5f-5i).

To further investigate how ASF1B regulates ENO1 activity, molecular docking analysis was performed and suggested that ASF1B directly bound to ENO1 (Fig. 5f). Structural modeling identified key hydrogen bonds between the N-terminal core domain of ASF1B and specific residues of ENO1. To experimentally validate this interaction, a series of ASF1B truncation mutants were generated: Flag-1# encoded full-length ASF1B, Flag-2# contained the N-terminal fragment (1–156 amino acids [aa]), and Flag-3# contained the C-terminal fragment (156–202 aa). Co-IP assays showed that Flag-2# interacted with ENO1, whereas Flag-3# did not, indicating that ENO1 bound to the N-terminal core region of ASF1B (Fig. 5g). Consistently, enzymatic assays of cell lysates demonstrated that ENO1 activity was significantly increased in the Flag-2# group, but no notable change was detected in the Flag-3# group relative to the sh-control group (Fig. 5h). Together, these results demonstrated that ASF1B bound to ENO1 through its N-terminal core region and enhances ENO1 enzymatic activity.

Previous studies have identified ENO1 as an important activator of the PI3K/Akt signaling pathway [29–35]. We therefore investigated whether ILA-induced downregulation of ASF1B affects this pathway (Fig. 5i). WB analysis showed that neither ILA treatment nor ASF1B knockdown altered total protein levels of PI3K/Akt pathway components, but both significantly reduced the phosphorylation levels of PI3K and Akt. The phosphorylation of downstream effectors, including mTOR and NF-κB, was also markedly decreased. In addition, key regulatory proteins were significantly altered, including the anti-apoptotic proteins Mcl-1 and Bcl-2, the pro-apoptotic protein Bax, the proliferation-associated protein Cyclin D1, and epithelial-mesenchymal transition (EMT) markers Vimentin and E-cadherin. These molecular changes were consistent with suppressed proliferation and invasion and increased apoptosis. These findings indicate that ILA-mediated downregulation of ASF1B inhibits ENO1 enzymatic activity, thereby suppressing PI3K/Akt signaling.

### ILA/ASF1B remodeled the TME of PCa through modulation of CXCL8

**Fig. 6:**
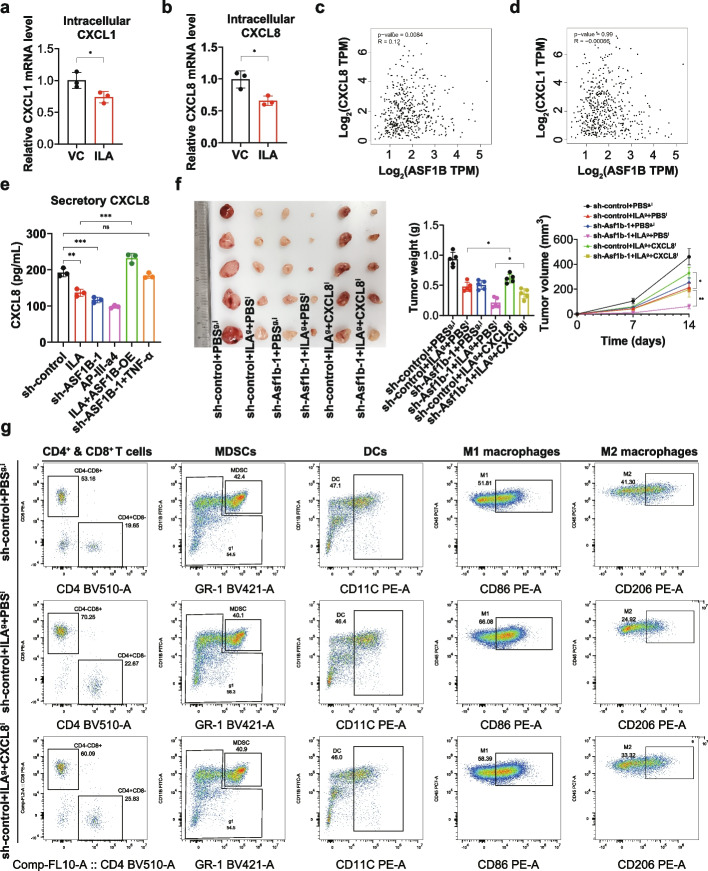
ILA remodeled the TME of PCa. **a** RNA-seq analysis of PC3 cells revealed decreased intracellular CXCL1 levels in the ILA group. **b** RNA-seq analysis of PC3 cells revealed decreased intracellular CXCL8 levels in the ILA group. **c** Positive correlation between ASF1B and CXCL8 based on GEPIA (PCa dataset). **d** No significant correlation between ASF1B and CXCL1 based on GEPIA (PCa dataset). **e** Secretory CXCL8 levels detected in DU145 cells under different treatment conditions. **f** C57BL/6 J mouse subcutaneous syngeneic tumor models with RM1 cells. ^g^gavage; ^i^intratumoral injection. Student’s t-test was used for two groups. Multiple group comparisons were performed using ANOVA, and then Tukey’s post hoc test was employed. **g** Impact of ILA on six immune cell subsets in C57BL/6 J syngeneic tumor models. Each experiment was repeated three times. Data are expressed as mean ± SD (*n* = 3–5). Statistical comparisons were performed using Student’s t-test for two groups and one-way ANOVA followed by Tukey’s post hoc test for multiple groups. TME, tumor microenvironment

Given that NF-κB regulates the expression of multiple chemokines [36], we focused on chemokines that were significantly altered in our RNA-seq dataset. Among these, CXCL1 and CXCL8 were markedly downregulated in the ILA-treated group (Fig. 6a and b, *p* < 0.05). Correlation analysis using GEPIA further showed that ASF1B expression was positively correlated with CXCL8 (Fig. 6c, *R* = 0.12, *p* < 0.01), whereas no significant correlation was observed with CXCL1 (Fig. 6d, *p* > 0.05). As chemokines are typically secreted to exert their biological functions [37], we next measured CXCL8 levels in culture supernatants. Compared with the sh-control group, CXCL8 secretion was significantly reduced following treatment with ILA, sh-ASF1B, or AP-III-a4 (Fig. 6e, *p* < 0.001). Moreover, ASF1B overexpression reversed the ILA-induced decrease in CXCL8 secretion, whereas treatment with TNF-α, an NF-κB agonist, restored CXCL8 levels suppressed by ASF1B knockdown. These findings suggest that CXCL8 is a key downstream chemokine regulated by the ILA/ASF1B/ENO1/PI3K/Akt/NF-κB signaling axis in PCa.

To further investigate the role of the ILA/ASF1B/CXCL8 axis in the TME, stable Asf1b-knockdown RM1 cell lines were generated (Fig. S6a) and used to establish subcutaneous syngeneic tumors in C57BL/6 J mice. Both ILA treatment and Asf1b knockdown significantly inhibited tumor growth (Fig. 6f). Notably, administration of CXCL8 together with ILA significantly increased tumor volume and weight compared with ILA treatment alone (Fig. 6f, short hairpin control RNA [sh- control] + ILA + phosphate-buffered saline [PBS] *vs.* sh-control + ILA + CXCL8, *p* < 0.05; sh-Asf1b + ILA + PBS *vs.* sh-Asf1b + ILA + CXCL8, *p* < 0.01). Mice lack the endogenous gene encoding CXCL8 [38]. Although human CXCL8 can bind to murine CXCR1/2, its agonistic efficiency for these receptors is relatively weak [39]. In contrast, murine CXCL1 is a high‑affinity ligand for murine CXCR2 and can effectively activate the CXCR1/2 signaling axis. Therefore, CXCL8 is often functionally replaced by CXCL1 in mouse model studies [40]. Given that ILA/ASF1B downregulates the expression of both CXCL1 and CXCL8, we further performed intratumoral injection of murine CXCL1 for validation. Consistent with the above findings, supplementation with murine CXCL1 markedly reversed the tumor-suppressive phenotype induced by Asf1b knockdown (Fig. S6b). These results suggest that CXCL8 partially attenuates the antitumor effect of ILA, likely through modulation of immune cell infiltration within the TME.

To characterize these immune changes, tumor tissues from three groups (sh-control + PBS, sh-control + ILA + PBS, and sh-control + ILA + CXCL8) were analyzed by flow cytometry (FC). As shown in Fig. 6g and Fig. S6c and 6d, ILA treatment significantly increased the proportions of infiltrating CD8^+^ T cells and M1 macrophages but diminished the number of M2 macrophages (*p* < 0.05), indicating enhanced CD8^+^ T cell infiltration and macrophage M1 polarization. In contrast, co-treatment with CXCL8 markedly reduced the fractions of CD8^+^ T cells and M1 macrophages and increased the proportion of M2 macrophages compared with ILA treatment alone. These results indicate that ILA exerts antitumor effects by suppressing CXCL8 expression, thereby promoting CD8^+^ T cell infiltration and macrophage M1 polarization and ultimately inhibiting PCa progression. Notably, ILA had no significant effects on the infiltration of CD4^+^ T cells, MDSCs, or dendritic cells (DCs) (Fig. S6d).

**Fig. 7:**
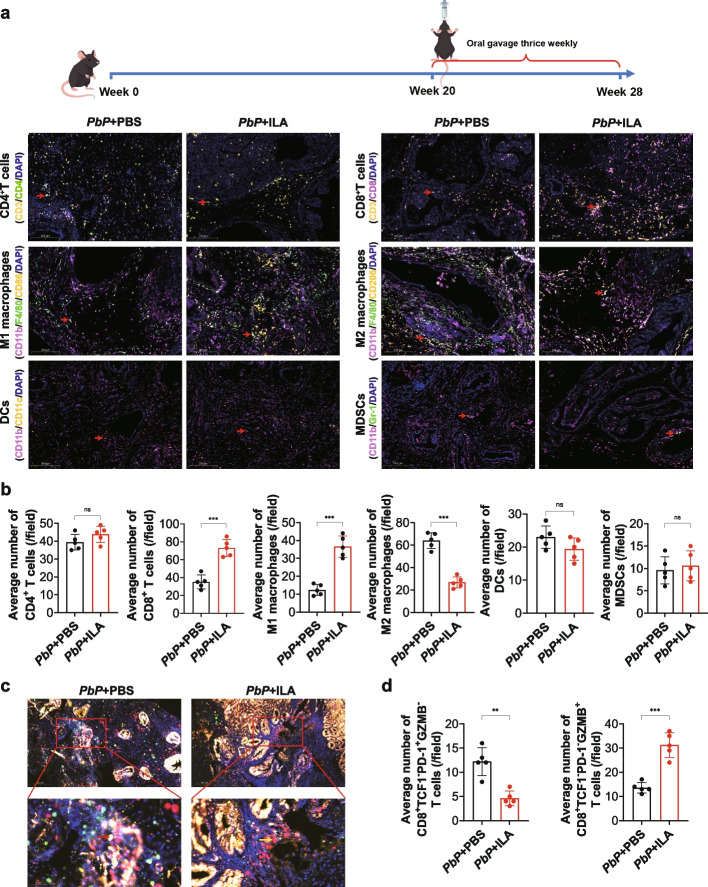
Impact of ILA on six immune cell subsets in *Pb-Cre4*; *Pten*^*flox/flox*^ PCa models. **a** Representative multicolor immunofluorescence plots of immune cell subsets. **b** Quantification of Fig. 7a. **c** Representative multicolor immunofluorescence plots of CD8^+^ T cell subsets. Red: CD8; white: TCF1; green: PD-1; yellow: GZMB; blue: DAPI. Red arrow indicates exhausted T cells (CD8^+^TCF1^−^PD-1^+^GZMB^−^), and yellow arrow indicates effector T cells (CD8^+^TCF1^−^PD-1^−^GZMB^+^). **d** Quantification of Fig. 7c. Data are expressed as mean ± SD (*n* = 5). Statistical comparisons were performed using Student’s t-test for two groups. ns, no statistical difference

Finally, we further validated the tumor-suppressive effect of ILA and its regulation of the TME in *PbP* mice. Hematoxylin and eosin (H&E) staining showed severe atypia, abundant mitoses and comedonecrosis in the PBS group, which were alleviated after ILA treatment (Fig. S7a). The lower Ki-67 proliferation index in the ILA group confirmed that ILA restrains tumor growth in *PbP* mice (Fig. S7b). Consistent with the syngeneic model, ILA significantly altered the infiltration of CD8^+^ T cells and macrophage subsets (M1 and M2), but did not affect CD4^+^ T cells, MDSCs, or DCs (Fig. 7a and 7b). Through further subset analysis of CD8^+^ T cells, we found that the increased infiltration was primarily composed of effector T cells (CD8^+^TCF1^−^PD-1^−^GZMB^+^), whereas the infiltration of exhausted T cells (CD8^+^TCF1^−^PD-1^+^GZMB^−^) was reduced (Fig. 7c and d). Collectively, these findings demonstrate that the ILA/ASF1B axis reprograms the PCa TME through modulation of CXCL8.

### ILA enhanced the efficacy of anti-PD-1 therapy

**Fig. 8:**
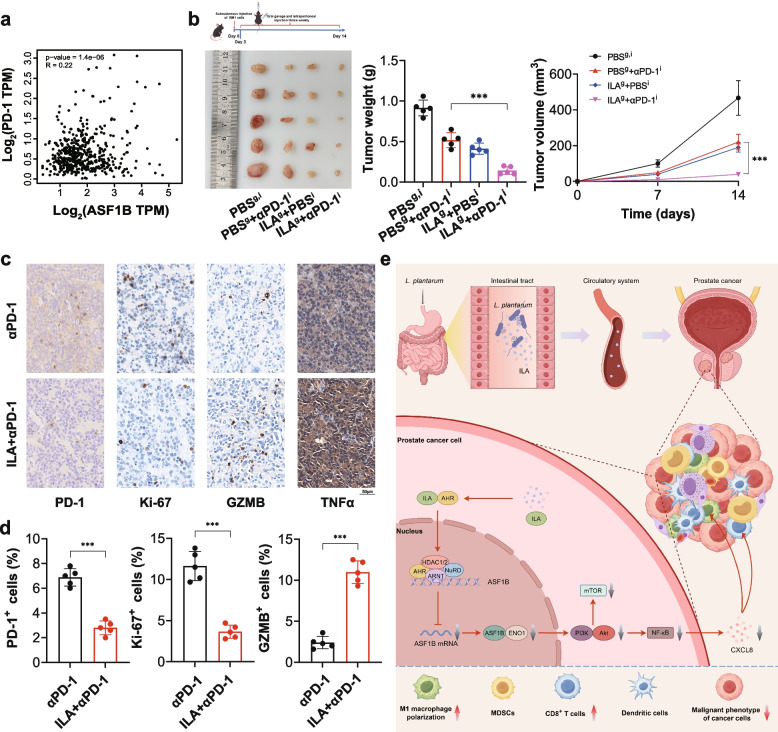
Synergistic suppression effects of ILA plus αPD-1 and key signaling pathways. **a** Positive association of ASF1B with PD-1 based on GEPIA (PCa dataset, *n* = 498). **b** C57BL/6 J mouse subcutaneous syngeneic tumor models with RM1 cells. ^g^gavage; ^i^intraperitoneal injection. **c** Representative IHC images of PD-1, Ki-67, GZMB, and TNFα expression. **d** Quantification of Fig. 8c. TNFα shows moderate expression in the αPD-1 group and strong expression in the αPD-1 plus ILA group. **e** Schematic model of ILA-mediated inhibitory mechanism against PCa. Each experiment was repeated three times. Data are expressed as mean ± SD (*n* = 5). Statistical comparisons were performed using Student’s t-test for two groups and one-way ANOVA followed by Tukey’s post hoc test for multiple groups. αPD-1: anti-PD-1 antibody; GZMB, granzyme B; ILA, indole-3-lactic acid

Analysis of GEPIA dataset revealed a significant positive correlation between ASF1B and PDCD1 (PD-1) expression (Fig. 8a, *r* = 0.22, *p* < 0.001). Based on this observation, we hypothesized that ILA might synergize with anti-PD-1 immunotherapy. In vivo experiments demonstrated that combined treatment with anti-PD-1 and ILA produced significantly greater tumor suppression than either ILA or anti-PD-1 monotherapy (Fig. 8b, *p* < 0.001). Further IHC analysis demonstrated that the combined treatment markedly decreased the levels of PD-1 and the proliferation marker Ki-67, while increasing the levels of granzyme B (GZMB) and TNF-α (Fig. 8c and d). Together, these findings indicate that ILA enhances the therapeutic efficacy of anti-PD-1 immunotherapy in PCa.

## Discussion

Studies exploring the GM in extra-gastrointestinal malignancies remain limited. In the present study, using both in vitro and in vivo experiments, we demonstrated that *L. plantarum*-derived ILA suppressed PCa progression through dual mechanisms (Fig. 8e). First, ILA inhibited the malignant behaviors of PCa cells via the ASF1B/ENO1/PI3K signaling axis. Second, ILA decreased CXCL8 secretion by suppressing NF-κB signaling, thereby remodeling the TME and further restraining PCa progression. In addition, we found that ILA enhanced the efficacy of anti-PD-1 immunotherapy. Collectively, these findings revealed a functional link between the GM and PCa and highlight the therapeutic potential of ILA for PCa management.

As a member of the H3-H4 histone chaperone family, ASF1B has been reported in prior studies to be frequently overexpressed and exhibits pro‐oncogenic functions across multiple cancers, including PCa [27, 41–43]. Here, we revealed for the first time the mechanism by which ILA downregulated ASF1B and comprehensively assessed its function in PCa. ILA bound to the AHR, inducing a conformational change that promoted nuclear translocation. Within the nucleus, AHR formed a heterodimer with ARNT, which specifically bound to the DRE sites in the ASF1B promoter, recruiting the HDAC1/2-NuRD corepressor complex. This recruitment decreased H3K27ac levels, leading to chromatin condensation and transcriptional repression of ASF1B.

In our study, ENO1 was demonstrated to be a crucial downstream regulator mediating the effects of ASF1B. As documented in previous studies, ENO1 is a key glycolytic enzyme that plays a critical role in the energy metabolism of tumor cells [44]. Beyond its canonical role in glycolysis, ENO1 has multifaceted functions, including regulation of specific mRNA translation, iron metabolism, and immune responses [45–47]. ENO1 is frequently upregulated in various malignancies and is associated with poor prognosis [48–50]. Interestingly, in PCa, we observed no significant upregulation of ENO1 mRNA. Instead, the antitumor effects of ILA/ASF1B were primarily mediated through suppression of ENO1 enzymatic activity rather than changes in its expression levels.

Beyond its direct effects on PCa cells, we found that ASF1B/ENO1 also modulates immune cell infiltration within the TME. Pan-cancer bioinformatic analyses by Ma et al. [51] revealed that ASF1B may influence tumor progression by altering the proportions of macrophages and Tregs in the TME. Similarly, other studies reported significant correlations between ASF1B expression and immune cell infiltration in hepatocellular carcinoma [52]. However, these investigations were limited to computational analyses. In this study, we experimentally demonstrated for the first time that the ILA/ASF1B axis remodeled the PCa TME by regulating the chemokine CXCL8.

CXCL8 has garnered increasing attention for its role in the TME. It acts not only as a signaling mediator among tumor cells, TAMs, and other immune cells, but also critically regulates tumor aggressiveness, metastasis, and angiogenesis [53]. Previous studies have reported that CXCL8 was elevated in PCa and closely correlated with advanced clinical stages [54]. Maynard et al. [55] further demonstrated that upregulated CXCL8 expression was associated with reduced androgen receptor (AR) levels and positively correlated with PCa severity, suggesting that CXCL8 may serve as a biomarker of PCa progression. Consistently, in our study, mice receiving combined ILA and CXCL8 treatment exhibited significantly larger tumor volumes compared with those treated with ILA alone, aligning with these prior observations.

This study demonstrates that ILA derived from *L. plantarum* is a potent tumor suppressor. Although lactate and ILA share a structural derivative relationship, they exert completely distinct biological functions in regulating tumor progression and immune homeostasis. As a core metabolite of the Warburg effect, lactate acidifies the TME and drives immunosuppressive reprogramming of immune cells, leading to tumor immune escape [56–58]. Lactate also serves as a substrate for histone lysine lactylation, which remodels epigenetic landscapes and activates genes related to metastasis, angiogenesis and drug resistance, further exacerbating tumor malignancy [59]. This marked functional difference between lactate and ILA further highlights the unique therapeutic potential of ILA among microbial metabolites.

This study has several limitations. First, ASF1B interacts with multiple proteins, and the roles of additional interacting partners warrant further investigation. Second, only six major immune cell subtypes within the TME were analyzed; other populations, such as B cells and NK cells, will be explored in future studies. Third, we confirmed that ILA directly acts on AHR in PCa cells and further regulates the downstream ASF1B/CXCL8 signaling pathway. Nevertheless, studies have demonstrated that AHR is also widely expressed in various immune cell subsets [60–64]. We cannot completely rule out the possibility that AHR signaling in immune cells plays an auxiliary role in this process. Fourth, compared to advanced PCa, the mechanism underlying the upregulation of *L. plantarum* and ILA in early-stage PCa remains unclear. Fifth, given that PCa is characterized by a highly immunosuppressive TME, pronounced tumor heterogeneity, and complex molecular profiles, anti-PD-1 monotherapy achieves only limited overall efficacy in clinical practice [65]. Our animal experiments confirmed that ILA markedly enhances the anti-tumor effect of anti-PD-1 antibodies. However, substantial differences between mouse models and human patients may pose challenges to clinical translation.

## Conclusions

In summary, we revealed a novel mechanism by which GM-derived metabolites regulated PCa progression and immunometabolism, positioning ILA as a potential therapeutic agent to improve PCa treatment.

## Materials and methods

### Sample collection and 16S rRNA sequencing

The study was approved by the Ethics Committee of Shanghai Tenth People’s Hospital (SHSY-IEC-4.1/20–22/01). Written informed consent was obtained from all participants for the scientific use of their fecal and tumor samples. Inclusion criteria were as follows: 1) patients aged ≥ 18 years; 2) patients undergoing prostate-specific antigen testing and abdominal ultrasound examination; and 3) Patients with the diagnosis of PCa confirmed by preoperative biopsy and postoperative paraffin pathology. Exclusion criteria included: 1) prior ADT, radiotherapy, chemotherapy, or other cancer treatments; 2) history of other malignancies or relevant treatments within the past two years; 3) contraindications to prostate biopsy, such as coagulation disorders or urogenital infections; 4) previous prostate biopsy or prostate-related surgeries; 5) use of systemic antibiotics, immunosuppressants, or other interventions affecting fecal microbiota within one month; and 6) patients under guardianship, supervision, or otherwise unable to provide informed consent. 16S rRNA sequencing was carried out by Annuoyouda Biotechnology Co., Ltd., Zhejiang, China.

### Cell culture

The PCa cell lines DU145, PC3, LNCaP, and RM1 were obtained from the Cell Resource Center, Shanghai Institute of Life Sciences, Chinese Academy of Sciences (Shanghai, China), and maintained at the Department of Urology, Shanghai Tenth People’s Hospital. Cells were cultured in RPMI 1640 medium supplemented with 10% fetal bovine serum (FBS) and 1% penicillin/streptomycin (complete medium). To control for solvent effects, the VC group was treated with DMSO at the same final concentration (0.08%) as used in the ILA treatment group (Aladdin, I157602) group.

### Cell proliferation assays

Cell proliferation was assessed using the CCK8 assay. Cells were seeded at 3,000 cells per well in a 96-well plate with three replicates per condition. Optical density at 450 nm was measured using a microplate reader. Each experiment was repeated three times.

### Plate clone formation assay

Colony-forming capacity was evaluated using the plate clone formation assay. Cells were seeded at a density of 1,000 cells per well in a 6-well plate and cultured for 14 days. Colonies were subsequently fixed, stained, and counted to assess clonogenic potential. Each experiment was repeated three times.

### Wound-healing assay

Cell migration was evaluated using the wound-healing assay. A uniform scratch was created in a confluent cell monolayer, and wound closure was monitored by microscopy. Each experiment was repeated three times.

### Transwell invasion assay

Cell invasive capacity was assessed using Transwell invasion assay. Briefly, 5 × 10^4^ cells were seeded to each Transwell chamber. Invaded cells were visualized using a fluorescence microscope (Olympus, Japan) and quantified with ImageJ software for statistical analysis. Each experiment was repeated three times.

### 3D spheroid invasion assay

To further evaluate cell invasive capacity, a 3D spheroid invasion assay was performed [66]. Images were captured using a fluorescence microscope and analyzed with ImageJ software. Each experiment was repeated three times.

### Apoptosis assay

Cell apoptosis was assessed using the Apoptosis Detection Kit (Vazyme, A211-01). The proportions of apoptotic cells were quantified with FlowJo software (version 10.0). Each experiment was repeated three times.

### Preparation of the bacteria suspension

*L. plantarum* were cultured in de Man, Rogosa and Sharpe (MRS) broth at 37 °C under anaerobic conditions. The bacterial culture was then diluted in sterile PBS to a concentration of 1 × 10^1^⁰ colony-forming units (CFUs)/mL for oral gavage.

### IHC

IHC was performed as previously described [67]. Detailed antibody information is provided in Table S4.

### PCR

qRT-PCR was performed using a one-step RT-PCR Kit (Qiagen, CA) according to the manufacturer’s protocol. Primer sequences were as follows: (1) GAPDH: forward 5'-GCCAAGGTCATCCATGACAACTTTGG-3', reverse 5'-GCCTGCTTCACCACCTTCTTGATGTC-3'; (2) ASF1B: forward 5'-TCCGGTTCGAGATCAGCTTC-3', reverse 5'-GTCGGCCTGAAAGACAAACA-3'. Real‑time PCR was performed to quantify total fecal bacteria using universal 16S rRNA gene primers (341F: 5′‑CCTACGGGAGGCAGCAG‑3′; 534R: 5′‑ATTACCGCGGCTGCTGG‑3′) and to detect *L. plantarum* using specific primers (F: 5′‑CCGTTATGCGGAACACCTA‑3′; R: 5′‑TCGGATTACCAAACATCAC‑3′). Each experiment was repeated three times.

### WB

WB was performed as previously described [68]. Detailed antibody information is provided in Table S4. Each experiment was repeated three times.

### Knockdown and overexpression cell line construction

Lentiviral vectors carrying human sh-ASF1B or sh-control were purchased from HANBio (Shanghai, China). Detailed sequences are listed in Table S5. The mouse sh-Asf1b-1 sequence (GTGGGCTACTATGTCAACAAT) was obtained from Sigma-Aldrich, and an additional sh-Asf1b-2 plasmid (sc-72564-SH) was purchased from Santa Cruz to reduce off-target effects. For ASF1B overexpression, the pcDNA3.1-ASF1B plasmid (ASF1B-OE) was acquired from GENEray (Shanghai, China).

### Luciferase reporter assays

AhR Luciferase Reporter Plasmid (Yeason, 11537ES03) were used to generate PC3-Lucia AHR and DU145-Lucia AHR cell lines. HANBIO (Shanghai, China) designed and produced pGL3 luciferase vectors containing either wild-type or mutated DRE sequences within the ASF1B promoter region. Transfections were performed using Lipofectamine 2000 (Invitrogen). Firefly and Renilla luciferase activities were quantified using the dual-luciferase assay kit (Promega) complying with the manufacturer’s instructions. Each experiment was repeated three times.

### LC–MS, ChIPseq, and ChIP-qPCR

LC–MS data was obtained from Liu et al. [27], and ChIPseq data were retrieved from Cistrome DB (http://cistrome.org/db/). ChIP-qPCR was performed as previously described [69]. Antibodies used included anti-AHR (CST, 83200 T) and anti-H3K27ac (Abcam, ab4729). Primers targeting the ASF1B promoter were: forward 5'-CGCGCTGCGGGATGC-3' and reverse 5'-ACGCTCCCTGGCGGC-3'.

### Co-IP

Co-IP was performed using the Classic IP/Co-IP Kit (Thermo, USA) according to the manufacturer’s instructions. Detailed antibody information is provided in Table S4. Each experiment was repeated three times.

### In vivo experiments

All animal experiments were conducted complying with guidelines for the welfare and ethics of experimental animals of Shanghai Tenth People’s Hospital, and approved by its Animal Experimental Ethics Committee of Shanghai Tenth People’s Hospital (SHDSYY-2024–6915). Three mouse strains were used: Balb/c nude, C57BL/6 J, and *Pb-Cre4*; *Pten*^*flox/flox*^ (*PbP*) mice. For subcutaneous xenograft models in nude mice, 3 × 10⁶ PC3 cells in 100 µL PBS were injected into the right hind limb on day 0. From day 2, mice received oral gavage three times per week with 200 µL of PBS, *L. plantarum* (2 × 10⁹ CFU), or ILA (20 mg/kg/mouse), respectively. For C57BL/6 J syngeneic models, 5 × 10^5^ RM1 cells in 100 µL PBS were injected into the right hind limb on day 0. In the chemokine experiments (starting day 2), mice were administered 200 µL of PBS or ILA via oral gavage and 100 µL of PBS, human CXCL8 (1 µg/mouse, R&D Systems, 208-IL/CF), or murine CXCL1 (1 µg/mouse, R&D Systems, 453-KC) via intratumoral injection, three times per week. In the anti-PD-1 experiments (starting day 3), mice received 200 µL of PBS or ILA via oral gavage and 200 µL of PBS or 200 µg of anti-PD-1 antibody (αPD-1, J43, BE0033-2, BioXcell) via intraperitoneal injection, three times per week. Tumor size and body weight of mice were measured weekly. Mice were euthanized via cervical dislocation at the conclusion of the tests.

*PbP* mice, prostate-specific Pten conditional knockout mice, were kindly provided by the Center for Excellence in Molecular Cell Science, Shanghai Institute of Biochemistry and Cell Biology, Chinese Academy of Sciences. These mice were used to establish orthotopic models of PCa. Body weight was monitored weekly, and from 20 to 28 weeks of age, mice received PBS or ILA via oral gavage, followed by cervical dislocation. Tumors were then dissected for multi-color immunofluorescence staining.

### Multicolor immunofluorescence staining

Multicolor immunofluorescence staining was performed as previously described [70]. Detailed antibody information is provided in Table S4.

### FC

Immune cell subtypes were identified via FC as previously described [71, 72]. Detailed antibody information is provided in Table S4. Each experiment was repeated three times.

### Enzyme linked immunosorbent assay (ELISA)

ELISA was performed following the instructions provided by the manufacturer. CXCL8 and ILA levels were quantified using commercial kits from Aifang Bio (AFE002-96 T) and NebuEasy (NBE-236831), respectively. Each experiment was repeated three times.

### ENO1 enzymatic activity assay and inhibition

ENO1 activity was measured using the Human ENO1 Activity Assay Kit (Abcam, ab117994) following the manufacturer’s protocol. AP-III-a4, a small-molecule inhibitor of alpha-enolase, was used to suppress ENO1 activity [35, 73].

## Statistical analysis

Comparisons between two groups were conducted using Student’s t-test. For multiple groups, one-way analysis of variance (ANOVA) followed by Tukey’s post hoc test was applied. A *P* value < 0.05 was considered statistically significant. *p < 0.05; **p < 0.01; ***p < 0.001. Statistical analyses and graphical representations were performed using R software (v4.4.0) and GraphPad Prism (v9.0).

## Supplementary Information

Supplementary Material 1.

## Data Availability

The raw data of the 16S rRNA sequencing are deposited at DOI: 10.5281/zenodo.19162088.
