# Supplementary material for: Lactobacillus plantarum-derived indole-3-lactic acid inhibits prostate cancer progression through ASF1B/ENO1 axis and remodels the tumor microenvironment to enhance anti-PD-1 therapy
Source: Mol Biomed. 2026 Aug 6;7:129. doi: 10.1186/s43556-026-00540-2 (PMC13442808; doi:10.1186/s43556-026-00540-2)
Supplement: Supplementary file 1 [file 43556_2026_540_MOESM1_ESM.docx]

**Fig. S1** **a** Comparison of relative fecal abundance of *L. plantarum* between stage T1/2 and T3/4 (n = 16). **b** Relative fecal abundance of *L. plantarum* in low (3+3, 3+4) versus high (≥4+3) Gleason score subgroups (n = 16). **c** Serum ILA concentrations in PCa patients stratified by low and high Gleason scores (n = 16). **d** Top 10 most significantly down-regulated genes in PCa identified by mRNA analysis. **e** Impact of the top 10 down-regulated genes on OS. **f** Impact of the top 10 down-regulated genes on DFS. Each experiment was repeated three times. Data are expressed as mean ± SD. Statistical comparisons were performed using Student’s t-test for two groups. OS, overall survival; DFS, disease-free survival; PCa, prostate cancer.

**Fig. S2 a** qRT-PCR showing significantly increased ASF1B expression in the ILA-treated group. **b** Protein levels of ASF1B in six PCa samples from our hospital detected by WB. **c** CCK-8 assay. **d** Wound-healing assay. **e** Transwell invasion assay. Each experiment was repeated three times. Data are expressed as mean ± SD (n = 3). Statistical comparisons were performed using Student’s t-test for two groups and one-way ANOVA followed by Tukey’s post hoc test for multiple groups.

**Fig. S3 a** Predicted binding sites between AHR and ASF1B according to JASPAR. **b** DRE sequence (5'-CACGC-3') in the ASF1B promoter. **c** ChIPseq results from Cistrome database. AHR, aryl hydrocarbon receptor; DRE, dioxin response element.

**Fig. S4 a** Interference efficiency of three different interference fragments. **b** CCK-8 assay. **c** Wound-healing assay. **d** Transwell invasion assay. **e** Apoptosis assay. Each experiment was repeated three times. Data are expressed as mean ± SD (n = 3). Statistical comparisons were performed using one-way ANOVA followed by Tukey’s post hoc test for multiple groups. ASF1B-OE, ASF1B overexpression.

**Fig. S5 a** Changes in the relative abundance of *L. plantarum* in mouse feces before and after gavage with *L. plantarum*. **b** Changes in serum ILA concentration in mice before and after gavage with *L. plantarum*. **c** Intracellular colocalization of ASF1B and ENO1. **d** Comparison of ENO1 mRNA expression between PCa (n = 492) and normal tissues based on GEPIA. **e** Impact of ENO1 on OS and DFS based on GEPIA. **f** CCK-8 assay. **g** Wound-healing assay. **h** Transwell invasion assay. **i** Apoptosis assay. Each experiment was repeated three times. Data are expressed as mean ± SD (n = 3-5). Statistical comparisons were performed using Student’s t-test for two groups and one-way ANOVA followed by Tukey’s post hoc test for multiple groups. OS, overall survival; DFS, disease-free survival.

**Fig. S6 a** Interference efficiency of sh-Asf1b-1 and sh-Asf1b-2 in RM1 cells. **b** Murine CXCL1 supplementation abrogates the tumor-suppressive effect of Asf1b knockdown. **c** Representative gating strategies for FC analysis of immune cell subsets. **d** Quantification of Fig. 6g. Each experiment was repeated three times. Data are expressed as mean ± SD (n = 3). Statistical comparisons were performed using Student’s t-test for two groups and one-way ANOVA followed by Tukey’s post hoc test for multiple groups. ns, no statistical difference.

**Fig. S7 a** H&E staining of *PbP* tumors. **b** Ki‑67 IHC staining and statistical quantification of *PbP* tumors. Each experiment was repeated three times. Data are expressed as mean ± SD (n = 5). Statistical comparisons were performed using Student’s t-test for two groups.

**Supplementary Table 1. Baseline clinical information of patients undergoing 16S rRNA high-throughput sequencing.**

| **Clinical indicators** | **Stage T1/2 (n = 27)** | **Stage T3/4 (n = 73)** | ***P*-value** |
| --- | --- | --- | --- |
| **Age (range, median)** | 63-81, 69 | 61-86, 70 | > 0.05 ^a^ |
| **Pathological types** |  |  | > 0.05 ^b^ |
| Adenocarcinoma | 27 | 73 |  |
| Others | 0 | 0 |  |
| **Grade** |  |  | > 0.05 ^b^ |
| 1~3 | 13 | 30 |  |
| 4~5 | 14 | 43 |  |
| **PSA** |  |  | > 0.05 ^b^ |
| <10 ng/mL | 11 | 20 |  |
| ≥10 ng/mL | 16 | 53 |  |
| **N category** |  |  | > 0.05 ^b^ |
| 0 | 24 | 54 |  |
| 1 | 3 | 19 |  |
| **M category** |  |  | > 0.05 ^b^ |
| 0 | 0 | 0 |  |
| 1 | 27 | 73 |  |
| **Number of dissected lymph nodes (range, median)** | 0-11, 5 | 0-15, 6 | > 0.05 ^a^ |

^a^t-test; ^b^chi-square test or Fisher’s exact test.

**Supplementary Table 2. Proteins binding to ASF1B revealed by LC-MS.**

| **Gene** | **Full names** |
| --- | --- |
| ASF1B | Histone chaperone ASF1B |
| MDH2 | Malate dehydrogenase, mitochondrial |
| FGFR1OP | FGFR1 oncogene partner |
| PPP2R1A | Serine/threonine-protein phosphatase 2A 65 kDa regulatory subunit A alpha isoform |
| PRRC2A | Isoform 2 of Protein PRRC2A |
| RPS17 | 40S ribosomal protein S17 |
| FOXF1 | Fork head box protein F1 |
| DHX29 | ATP-dependent RNA helicase DHX29 |
| SSSCA1 | Sjoegren syndrome/scleroderma autoantigen 1 |
| ENO1 | Alpha-enolase |
| PCM1 | Pericentriolar material 1 protein |
| PRDX5 | Isoform Cytoplasmic peroxisomal of Peroxiredoxin-5, mitochondrial |
| RAVER1 | Ribonucleoprotein PTB-binding 1 |
| FMR1 | Isoform 4 of Synaptic functional regulator FMR1 |
| YBX3 | Isoform 2 of Y-box-binding protein 3 |
| HNRNPC | Heterogeneous nuclear ribonucleoproteins C1/C2 |
| ENO3 | Beta-enolase (Fragment) |
| LDHB | L-lactate dehydrogenase (Fragment) |
| CDK9 | Cyclin-dependent kinase 9 |
| CPS1 | Isoform 2 of Carbamoyl-phosphate synthase [ammonia], mitochondrial |
| DHX36 | ATP-dependent RNA helicase DHX36 (Fragment) |
| EEF1G | Elongation factor 1-gamma |
| GPI | Glucose-6-phosphate isomerase (Fragment) |
| IQSEC1 | IQ motif and SEC7 domain-containing protein 1 |

Note: from "Liu X, et al. ASF1B promotes cervical cancer progression through stabilization of CDK9. Cell Death Dis. 2020;11(8):705. doi: 10.1038/s41419-020-02872-5"

**Supplementary Table 3. Correlation analysis between ASF1B and its binding genes.**

| **Query** | **Statistic** | **P-value** | **FDR (BH)** | **Event_SD** | **Event_TD** |
| --- | --- | --- | --- | --- | --- |
| ASF1B | 1 | 1.00E-178 | 1.00E-174 | 497 | 497 |
| ENO1 | 0.3211897 | 2.17E-13 | 4.67E-12 | 497 | 497 |
| FGFR1OP | 0.3087759 | 1.93E-12 | 3.61E-11 | 497 | 496 |
| GPI | 0.2998575 | 8.75E-12 | 1.48E-10 | 497 | 497 |
| HNRNPC | 0.2287267 | 2.54E-07 | 1.81E-06 | 497 | 497 |
| MDH2 | 0.2116176 | 1.94E-06 | 1.15E-05 | 497 | 497 |
| DHX36 | 0.1581434 | 0.0004017 | 0.0014083 | 497 | 497 |
| RAVER1 | 0.1440166 | 0.0012848 | 0.0039652 | 497 | 497 |
| CDK9 | 0.0967349 | 0.0310689 | 0.0622839 | 497 | 497 |
| RPS17 | 0.0881319 | 0.0495715 | 0.0920418 | 497 | 497 |
| EEF1G | 0.0784558 | 0.0805776 | 0.1383865 | 497 | 497 |
| SSSCA1 | 0.0701132 | 0.1185108 | 0.1908029 | 497 | 497 |
| PRDX5 | 0.0494971 | 0.2707403 | 0.3733829 | 497 | 497 |
| ENO3 | 0.0271867 | 0.5453963 | 0.6469321 | 497 | 497 |
| CPS1 | 0.0213358 | 0.6351388 | 0.7254026 | 497 | 495 |
| PPP2R1A | -0.015274 | 0.7341053 | 0.8056947 | 497 | 497 |
| FMR1 | -0.05241 | 0.2435085 | 0.3428785 | 497 | 497 |
| PCM1 | -0.127012 | 0.0045697 | 0.0120009 | 497 | 497 |
| IQSEC1 | -0.155991 | 0.0004826 | 0.0016634 | 497 | 497 |
| DHX29 | -0.20091 | 6.37E-06 | 3.41E-05 | 497 | 497 |
| LDHB | -0.287423 | 6.59E-11 | 9.49E-10 | 497 | 497 |
| FOXF1 | -0.317506 | 4.20E-13 | 8.66E-12 | 497 | 497 |

**Supplementary Table 4. Antibody information.**

| **Experiments** | **Antibody** | **Company** | **Catalog number** |
| --- | --- | --- | --- |
| **WB** | PI3K | CST | 4257T |
|  | p-PI3K | CST | 4228T |
|  | AKT | CST | 4691T |
|  | p-Akt | CST | 4060T |
|  | NF-κB | CST | 4764T |
|  | p-NF-κB | CST | 3033T |
|  | mTOR | CST | 2972S |
|  | p-mTOR | CST | 2971S |
|  | Mcl-1 | CST | 4572S |
|  | Bcl-2 | CST | 3498T |
|  | Bax | CST | 2772T |
|  | Cyclin D1 | CST | 2922S |
|  | Vimentin | CST | 5741T |
|  | E-Cadherin | CST | 3195T |
|  | GAPDH | Abcam | ab181602 |
|  | Peroxidase-conjugated anti-mouse&anti-rabbit secondary antibody | AlpVHHs | 001-300-005 |
| **Co-IP** | SIN3A | Santa Cruz | sc-5299 |
|  | CHD4 | Abcam | ab70469 |
|  | RCOR1 | AmyJet | 75-039 |
|  | HDAC1 | CST | 5356 |
|  | AHR | CST | 83200T |
| **Multicolor immunofluorescence staining** | PD-1 | Abcam | ab214421 |
|  | GZMB | Cellsignal | 46890t |
|  | TCF1 | CST | 2203s |
|  | CD3 | Abcam | ab16669 |
|  | CD4 | Abcam | ab183685 |
|  | CD8α | Abcam | ab217344 |
|  | CD11b | Abcam | ab133357 |
|  | F4/80 | CST | 70076s |
|  | CD86 | CST | 19589s |
|  | Ly6g | Abcam | ab238132 |
|  | CD11c | CST | 97585s |
|  | CD206 | Shanghai Huilan | abb00004 |
| **Flow cytometry** | Brilliant Violet 421 anti-mouse Ly-6G/Ly-6C (Gr-1) | BioLegend | 108445 |
|  | BV510 Rat Anti-Mouse CD4 | BD Pharmingen | 563106 |
|  | PE/Cyanine7 anti-mouse CD45 | BioLegend | 103114 |
|  | BB515 Rat Anti-Mouse CD45 | BD Pharmingen | 564590 |
|  | FITC Rat Anti-Mouse CD45 | BD Pharmingen | 553079 |
|  | PE anti-mouse CD206 (MMR) | BioLegend | 141705 |
|  | PE anti-mouse CD8a | BioLegend | 100707 |
|  | Brilliant Violet 421 anti-mouse F4/80 | BioLegend | 123137 |
|  | Brilliant Violet 421 anti-mouse CD3 | BioLegend | 100227 |
|  | PE anti-mouse CD86 | BioLegend | 105105 |
|  | FITC anti-mouse/human CD11b | BioLegend | 101205 |
|  | PE anti-mouse CD11c | BioLegend | 117307 |
| **IHC** | ASF1B | Proteintech | 22258-1-AP |
|  | PD-1 | Abcam | ab214421 |
|  | TNFα | Proteintech | 60291-1-Ig |
|  | GZMB | Abcam | ab255598 |

**Supplementary Table 5. Primers used for shRNA sequences.**

| sh-NC lentiviral vector | Top strand | GATCCGTTCTCCGAACGTGTCACGTAATTCAAGAGATTACGTGACACGTTCGGAGAATTTTTTC |
| --- | --- | --- |
|  | Bottom strand | AATTGAAAAAATTCTCCGAACGTGTCACGTAATCTCTTGAATTACGTGACACGTTCGGAGAACG |
| sh-ASF1B-1 | Top strand | GATCCGCTGGAGTGGAAGATCATTTATCTCGAGATAAATGATCTTCCACTCCAGTTTTTTG |
|  | Bottom strand | AATTCAAAAAACTGGAGTGGAAGATCATTTATCTCGAGATAAATGATCTTCCACTCCAGCG |
| sh-ASF1B-2 | Top strand | GATCCGCGGCTGAGAGTGAGGAATTTGATCACTCGAGTGATCAAATTCCTCACTCTCAGCCGTTTTTTG |
|  | Bottom strand | AATTCAAAAAACGGCTGAGAGTGAGGAATTTGATCACTCGAGTGATCAAATTCCTCACTCTCAGCCGCG |
| sh-ASF1B-3 | Top strand | GATCCGCAGGGAGACACATGTTTGTCTTTCACTCGAGTGAAAGACAAACATGTGTCTCCCTGTTTTTTG |
|  | Bottom strand | AATTCAAAAAACAGGGAGACACATGTTTGTCTTTCACTCGAGTGAAAGACAAACATGTGTCTCCCTGCG |
